# Supplementary material for: Hospital at home: A systematic review of how medication management is conceptualised, described and implemented in practice—A study protocol
Source: PLoS One. 2023 Jan 26;18(1):e0268846. doi: 10.1371/journal.pone.0268846 (PMC9879423; doi:10.1371/journal.pone.0268846)
Supplement: Attachment — Submitted filename: Response to reviewers comments.docx [file pone.0268846.s002.docx]

**Response to reviewer comments**

# Hospital at home: a systematic review of how medication management is conceptualised, described and implemented in practice – a study protocol.

Sophie McGlen, Clare Crowley, Daniel Lasserson, Zahra AL Qamariat and Rosemary Lim*

We want to thank the reviewers for taking the time to review our manuscript. Please find our responses to the reviewers’ feedback below. Changes made are shown in red text in the tracked-change document.

**Reviewer 1:**

- *Have the authors described where all data underlying the findings will be made available when the study is complete. Can authors explain this point?*

We have clarified this in the manuscript in the ethics and dissemination section, pg 9, lines 229-230. We have added the following sentence: “The data used in the study are available from the corresponding author on request.”

**Reviewer 2:**

- *Some sections (eligibility criteria, search strategy, and risk of bias) are poorly explained. They need to be further clarified, maybe by employing tables and/or figures*

We have incorporated the PI(E)COS eligibility criteria into a table and clarified we will be using the UK Hospital at Home Society definition of Hospital@Home in the eligibility criteria section (see pg 6, lines 164 onwards). This section now reads as:

|  | Eligibility |
| --- | --- |
| Participants/population | anyone receiving hospital care meeting the UK H@H society H@H definition, at their usual place of residence. |
| Interventions(s), Exposure(s) | medication management in a H@H setting |
| Comparator | where relevant e.g. trial of different medication management approaches |
| Outcomes | Any |
| Study Design | Any |

Regarding risk of bias, we have edited this section to now read as (pg 8, lines 199-203):

“Two independent reviewers will use the Mixed Methods Appraisal Tool (MMAT) version 2018 (22) to critically appraise eligible studies available online at <http://mixedmethodsappraisaltoolpublic.pbworks.com/w/file/fetch/127916259/MMAT_2018_criteria-manual_2018-08-01_ENG.pdf> This tool allows the appraisal of quantitative, qualitative and mixed methods studies to reduce the risk of reviewer bias.”

- *Full search query should be also provided for reproducibility.*

We have included a table showing our search strategy that reads as (see pg 7):

The search strategy will include the following search terms:

| # | Searches |
| --- | --- |
| 1 | “hospital at home” |
| 2 | “hospital@home” |
| 3 | “hospital in the home” |
| 4 | “virtual ward” |
| 5 | “admission avoidance” |
| 6 | 1 or 2 or 3 or 4 or 5 |
| 7 | Pharmacist or clinical pharmacist |
| 8 | pharmac* |
| 9 | medication |
| 10 | “medication management” |
| 11 | “drug delivery” |
| 12 | “medicine optimization” |
| 13 | 7 or 8 or 9 or 10 or 11 or 12 |
| 14 | 6 AND 13 |
| 15 | Limit 14 to English language |

Searches will use Index Terms unique to each database and a combination of Boolean (AND/OR) keywords, as relevant. Grey literature will be identified using Google Scholar.

- *A final discussion section should be provided, highlighting advantages of conducting a systematic review on H@H topic, major limiting factors, and expectations.*

We have included a discussion section (see pg 10) at the end of the manuscript including the advantages and limitations of undertaking this systematic review. This section reads as:

“The H@H concept is a rapidly evolving healthcare service particularly in the UK where the implementation of virtual wards has been a key policy in the NHS post covid-19 recovery plans. It is viewed as an option to deliver healthcare without increasing demand on the traditional inpatient hospital setting. As medication delivery is critical to the H@H function, understanding the literature available will support implementation of future H@H services.

This review will draw on literature from a variety of healthcare systems worldwide. This will not always be applicable to the UK healthcare system and therefore will pose a limitation to the findings. Despite the increase in virtual wards within the UK, not all of these meet the UKH@H society definition. Only publications of services that meet this definition will be included within this review.”
